# The Correlation between Lung Sound Distribution and Pulmonary Function in COPD Patients

**DOI:** 10.1371/journal.pone.0107506

**Published:** 2014-09-22

**Authors:** Masamichi Mineshita, Hirotaka Kida, Hiroshi Handa, Hiroki Nishine, Naoki Furuya, Seiichi Nobuyama, Takeo Inoue, Shin Matsuoka, Teruomi Miyazawa

**Affiliations:** 1 Division of Respiratory and Infectious Diseases, Department of Internal Medicine, St. Marianna University School of Medicine, Kawasaki, Japan; 2 Department of Radiology, St. Marianna University School of Medicine, Kawasaki, Japan; Scientific Inst. S. Raffaele Hosp., Italy

## Abstract

**Background:**

Regional lung sound intensity in chronic obstructive pulmonary disease (COPD) patients is influenced by the severity and distribution of emphysema, obstructed peripheral airways, and altered ribcage and diaphragm configurations and movements due to hyperinflation. Changes in the lung sound distribution accompanied by pulmonary function improvements in COPD patients were observed after bronchodilator inhalation. We investigated the association of lung sound distribution with pulmonary functions, and the effects of emphysematous lesions on this association. These studies were designed to acquire the basic knowledge necessary for the application of lung sound analysis in the physiological evaluation of COPD patients.

**Methods:**

Pulmonary function tests and the percentage of upper- and lower-lung sound intensity (quantitative lung data [QLD]) were evaluated in 47 stable male COPD patients (54 - 82 years of age). In 39 patients, computed tomography taken within 6 months of the study was available and analyzed.

**Results:**

The ratio of lower QLD to upper QLD showed significant positive correlations with FEV_1_ %predicted (%FEV_1_; ρ = 0.45, p<0.005) and MEF_50_ %predicted (%MEF_50_; ρ = 0.46, p<0.005). These correlations were not observed in COPD patients with dominant emphysema (% low attenuation area >40%, n = 20) and were stronger in less emphysematous patients (n = 19, %FEV_1_; ρ = 0.64, p<0.005, %MEF_50_; ρ = 0.71, p<0.001).

**Conclusions:**

In COPD patients, the ratio of lower- to upper-lung sound intensities decreased according to the severity of obstructive changes, although emphysematous lesions considerably affected lung sound distribution.

## Introduction

Chronic obstructive pulmonary disease (COPD) is characterized by progressive airflow limitation. Because airflow in the lung produces sound, a generalized reduction in apparent lung sound is one of the indicators of COPD. A correlation between the perceived lung sound intensity and the percent-predicted forced expiratory volume over one second (FEV_1_) has been reported [1]. Although lung sounds are reported to be generated primarily within the lobar to segmental airways [2], Ploysongsang compared Xenon ventilation scans with the distribution of lung sound intensities and reported that regional lung sound intensity could be used to quantify regional ventilation in subjects with emphysema in whom the physiological deficits are mainly caused by small airway obstruction [3].

Regional lung ventilation in COPD patients seems to be influenced by altered ribcage and diaphragm configurations and movement due to hyperinflation. In severe COPD patients, the movements of the diaphragm are restricted, and accessory respiratory muscles, such as sternocleidomastoid and scalene muscles, are recruited during tidal breathing [4], [5]. In these circumstances, it seems that regional ventilation may shift from the lower- to upper-lung field, according to the degree of hyperinflation. Therapeutic interventions that improve hyperinflation in COPD are thought to modulate regional airflow in the lung, which is reflected by the changes in regional lung sound distribution.

Recently, improvements in computer technology have provided new insights into acoustic analysis and have been introduced in clinical studies as a surrogate marker of regional ventilation [6]–[12]. In a previous study, we observed changes in the lung sound distribution that was accompanied by pulmonary function improvements in COPD patients after inhalation of a short-acting bronchodilator [13]. In seven patients with homogeneous emphysema, the relative regional lung sound intensity decreased in the upper-lung field and increased in the lower-lung field after bronchodilator inhalation. We hypothesized that this result was caused by an increase in lower lung airflow, which might reflect an improvement in diaphragm movement. However, we also found that the bronchodilator-induced relative increase in the lower lung sound intensity was not observed in one patient with heterogeneous emphysema or in one non-emphysematous patient. The distribution of emphysematous lesions and the bronchial reactivity appeared to influence regional lung ventilation and the distribution of lung sounds after bronchodilator use.

Although the distribution of emphysematous lesions was thought to influence the lung sound distribution, the assessment of regional lung sound might be applicable as a non-invasive tool for evaluating the regional physiological characteristics of COPD patients. In this study, we investigated the association of lung sound distribution with pulmonary function in COPD patients and healthy subjects, with the goal of acquiring basic knowledge regarding the association of lung sound distribution with pulmonary function. Furthermore, we analyzed the influence of emphysematous lesions on the lung sound distribution in COPD patients.

## Methods

This study was performed at St. Marianna University Hospital (Kanagawa, Japan). The ethics committee of St. Marianna University Hospital (No1230) approved this study. Written informed consent was obtained from all of the participants.

### Study population

Forty-seven clinically stable male COPD patients, each with a smoking history of more than 20 pack-years, were recruited. Patients were excluded from the study if they had received clinical diagnoses of asthma or bronchiectasis. The patients were permitted to continue using their medications for COPD. We were unable to recruit any female COPD patients due to the small number of female COPD patients at our hospital.

Forty healthy male smokers were recruited for comparison. Volunteers were deemed healthy on the basis of their clinical history, a physical examination and spirometric findings. Because all subjects underwent annual health checks, including chest X-rays, chest X-rays were not performed to avoid extra irradiation exposure. Subjects whose history included abnormal chest X-ray findings from the previous year were excluded. Individuals with a history of chronic cardiopulmonary disease, surgical chest procedures or recent (within 6 months) respiratory tract infections were excluded.

### Pulmonary function tests

Spirometry was performed using a calibrated spirometer according to the American Thoracic Society guidelines. The forced vital capacity (FVC), FEV_1_, maximum expiratory flow (MEF) at 50% of FVC (MEF_50_) and MEF at 25% of FVC (MEF_25_) were recorded. The predicted values for spirometric measurements were derived from the guidelines of the Japanese Respiratory Society [14]. The predicted values for the single-breath nitrogen washout test and the single-breath total lung diffusion capacity (DLco) were derived from Buist et al. [15] and Roca et al. [16].

### Lung sound recording

The breath sounds were recorded using the VRIxp System (Deep Breeze, Ltd., Or-Akiva, Israel) during deep and regular breaths, and the percentage of regional lung sound energy (quantitative lung data [QLD]) was calculated as previously described [6], [7], [9], [13]. Just prior to breath sound recordings, a physician examined the patients using a stethoscope. VRI recordings were performed with 7-row arrays (40 active sensors in total) when the subjects′ height was 165 cm or higher. We used 6-row arrays (34 active sensors in total) when the subjects′ height was below 165 cm (26 COPD patients and 7 healthy volunteers). After recording, data were divided into upper (rows 1–2), middle (rows 3–4 in the 6-row arrays and rows 3–5 in the 7-row arrays) and lower (rows 5–6 in the 6-row arrays and rows 6–7 in the 7-row arrays) zones, and the QLD for each of these six zones was generated [17]. The signal data were band-pass filtered (100–250 Hz) to reduce interference generated by chest wall movement and heart sounds. At least three lung sound recordings from each subject were produced, and one acceptable VRI recording with the highest technical quality was chosen by a single investigator (M.M.) before QLD evaluation using the following criteria: (1) the recordings were free of artifacts; (2) the patient maintained a proper breathing cycle (3–4/12seconds); and (3) the recording reflected an adequate breathing intensity with the most consistent breathing pattern [17]. VRI measurements from 40 healthy male volunteers were also recorded for comparison.

### Multi-detector computed tomography

In 39 patients, multi-detector computed tomography (Aquilion 64, Toshiba Medical Systems, Otawara, Japan) taken within 6 months was available and used for analysis. The CT parameters for inspiratory scans were as follows: collimation, 0.5 mm; 120 kVp; 200 mA; gantry rotation time, 0.5 sec; and beam pitch, 0.83 (table feed per gantry, 53; collimation beam width, 64). All images were reconstructed using a standard algorithm, with a slice thickness of 1 mm and a reconstruction interval of 0.5 mm. Three CT slices were selected for each subject; the upper cranial slice was obtained 1 cm above the upper margin of the aortic arch, the middle slice was obtained 1 cm below the carina, and the lower caudal slice was obtained 1 cm below the right inferior pulmonary vein. These CT images were then analyzed using a semiautomatic image processing program (Image J version 1.40 g, a public domain Java image-processing program available at http://rsb.info.nih.gov/ij/) as previously described [13]. Low-attenuation areas (LAA) between −950 to −1024 HU were identified as emphysema, and the percentage of the LAA for the entire lung area (%LAA) was calculated for both right and left sides of the upper, middle, and lower lung fields. In this study we defined emphysema-dominant COPD as an average regional %LAA of more than 40%.

### Statistical analysis

The data are reported as the mean ± standard deviation unless otherwise indicated. All of the analyses were performed using SPSS software (ver19; IBM, Armonk, NY, USA). The upper, middle, and lower QLD of COPD patients and healthy subjects were compared using a Mann-Whitney U test. The correlations between the lower QLD/upper QLD ratio and the spirometric measurements were evaluated using Spearman's rank correlation coefficient. A p-value of <0.05 was considered to be statistically significant.

## Results

Between April 2007 and March 2013, acceptable VRI recording data were obtained for 47 COPD patients. Recruited patients were free of respiratory infection and COPD exacerbation for at least 4 weeks prior to VRI recordings. Neither the radiograms nor the CT scans showed central airway obstructive lesions in these patients. A stethoscope examination performed before breath sound recordings revealed no wheezing for patients. The demographics, anthropometric values, and lung function test results of recruited COPD patients and healthy smokers are shown in Table 1. One patient was classified as GOLD I, 17 patients as GOLD II, 23 patients as GOLD III, and 6 patients as GOLD IV.

**Table 1:** Characteristics of subjects.

		COPD	Healthy smokers
Subjects		47 (Male)	40 (Male)
Mean age (y)		71.6±6.3***	40.0±15.1
Pack-Years		81.9±36.5***	18.3±18.0
Duration of exposition to smoke (y)		47.1±6.0***	15.8±9.8
BMI		22.6±3.4	23.7±2.4
Pulmonary function tests			
	FVC %predicted	88.1±18.2%	104.3±10.2%
	FEV_1_ %predicted	48.2±17.2%***	99.1±11.5%
	FEV_1_/FVC (%)	43.7±11.0%***	82.2±4.7%
	MEF_50_ %predicted	17.3±10.0%***	96.1±27.3%
	MEF_25_ %predicted	20.4±8.2%***	75.2±22.4%
	deltaN_2_ %predicted	556.4±274.4% (n = 32)	
	DLco %predicted	37.4±13.6% (n = 32)	
GOLD I		1	
GOLD II		17	
GOLD III		23	
GOLD IV		6	

Definition of abbreviations: BMI  =  body mass index, FVC  =  forced vital capacity, FEV_1_  =  forced expiratory volume in 1 second, MEF_50_ and MEF_25_  =  maximum expiratory flow at 50% and 25% of FVC, deltaN_2_  =  the phase III slope of the single-breath nitrogen washout test, DLco  =  the single-breath total lung diffusion capacity. Values are represented as mean ± standard deviation. ***; P<0.001 compared with healthy smokers.

**Figure 1:**
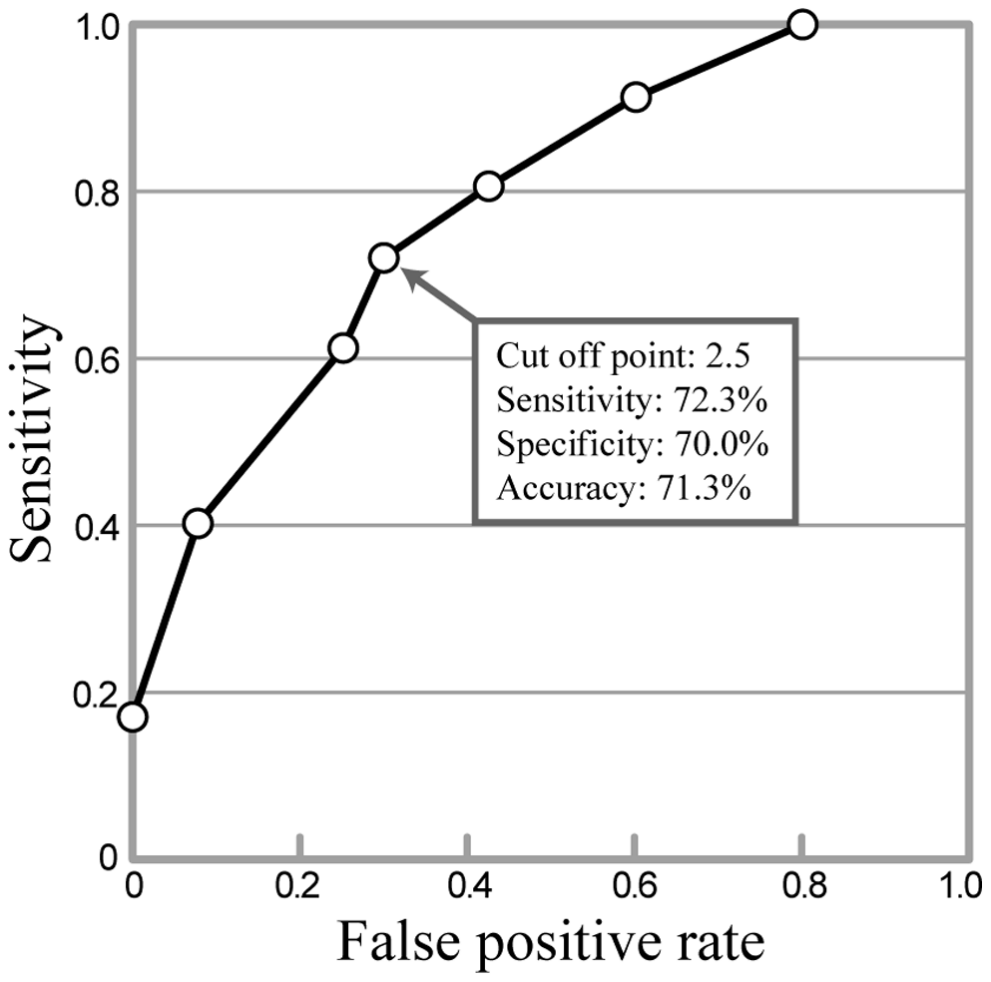
Receiver operating characteristic (ROC) validation of Lower QLD/Upper QLD. ROC validation of Lower QLD/Upper QLD revealed that a cut-off point of 2.5 yielded 72.3% sensitivity, 70% specificity, and 71.3% accuracy in the differentiation of COPD patients and healthy smokers.

In the COPD patients, the upper QLD was significantly higher and the lower QLD was significantly lower than the corresponding values in healthy male smokers. As a result, the ratio of the lower QLD to upper QLD (Lower QLD/Upper QLD) in COPD patients was approximately 60% of the ratio for the healthy male smokers (Table 2). Receiver-operating characteristics (ROC) validation of Lower QLD/Upper QLD revealed that using a cut-off point of 2.5 yielded 72.3% sensitivity, 70% specificity, and 71.3% accuracy in the differentiation of COPD patients and healthy smokers (Figure 1). According to the effect of age on Lower QLD/Upper QLD in healthy subjects, there was no significant difference in Lower QLD/Upper QLD between younger subjects (<40 years old, n = 24, mean age; 30.5±5.1 years, mean pack-years; 8.8±7.7, Lower QLD/Upper QLD; 2.9±1.1) and older subjects (≥40 years old, n = 16, mean age; 54.1±14.2 years, mean pack-years; 33.4±19.5, Lower QLD/Upper QLD; 3.52±1.33).

**Table 2:** Quantitative lung data (QLD) in COPD patients and healthy smokers.

	COPD (n = 47)	Healthy smokers (n = 40)
Upper	22.55±6.80%***	15.92±4.18%
Middle	39.75±8.30%	38.15±5.94%
Lower	37.70±10.24%***	45.92±8.02%
Lower QLD/Upper QLD	1.91±0.94***	3.18±1.24

Values are represented as mean ± standard deviation. ***; P<0.001 compared with healthy smokers.

The relationship between Lower QLD/Upper QLD and obstructive changes in the pulmonary function tests measurements were studied in COPD patients and healthy subjects (Table 3). Although there were no significant correlations between Lower QLD/Upper QLD and pulmonary functions in healthy subjects (Table 3), there were significant positive correlations for FEV_1_%predicted (%FEV_1_), FEV_1_/FVC, and MEF_50_ %predicted (%MEF_50_) in COPD patients (Table 3). In 32 COPD patients for whom the results of the single-breath nitrogen washout test and DLco were obtained, Lower QLD/Upper QLD showed a significant negative correlation with the phase III slope of the single-breath nitrogen washout test%predicted (deltaN_2_ %predicted), although there was no significant correlation between this ratio and DLco %predicted (Table 3).

**Table 3:** Relationship between Lower QLD/Upper QLD and pulmonary function tests.

	Lower QLD/Upper QLD			
	Normal (n = 40)	COPD (n = 47)	COPD %LAA <40% (n = 19)	COPD %LAA >40% (n = 20)
FVC %predicted	ρ = 0.11 (NS)	ρ = 0.25 (NS)	ρ = 0.27 (NS)	ρ = 0.36 (NS)
FEV_1_ %predicted	ρ = −0.06 (NS)	ρ = 0.45 (p<0.005)	ρ = 0.65 (P<0.005)	ρ = 0.24 (NS)
FEV_1_/FVC	ρ = −0.03 (NS)	ρ = 0.42 (p<0.005)	ρ = 0.74 (p<0.001)	ρ = −0.21 (NS)
MEF_50_ %predicted	ρ = −0.07 (NS)	ρ = 0.46 (p<0.005)	ρ = 0.71 (p<0.001)	ρ = 0.19 (NS)
MEF_25_ %predicted	ρ = −0.13 (NS)	ρ = 0.19 (NS)	ρ = 0.36 (NS)	ρ = 0.07 (NS)
delta N_2_ %predicted	-	ρ = −0.52 (p<0.005, n = 32)	-	-
Dlco %predicted	-	ρ = −0.11 (NS, n = 32)	-	-

Definition of abbreviations: FVC  =  forced vital capacity, FEV1  =  forced expiratory volume in 1 second, MEF_50_ and MEF_25_  =  maximum expiratory flow at 50% and 25% of FVC, deltaN_2_  =  the phase III slope of the single-breath nitrogen washout test, DLco  =  the single-breath total lung diffusion capacity, %LAA  =  the percentage of the Low-attenuation areas for the entire lung, NS  =  not significant.

**Figure 2:**
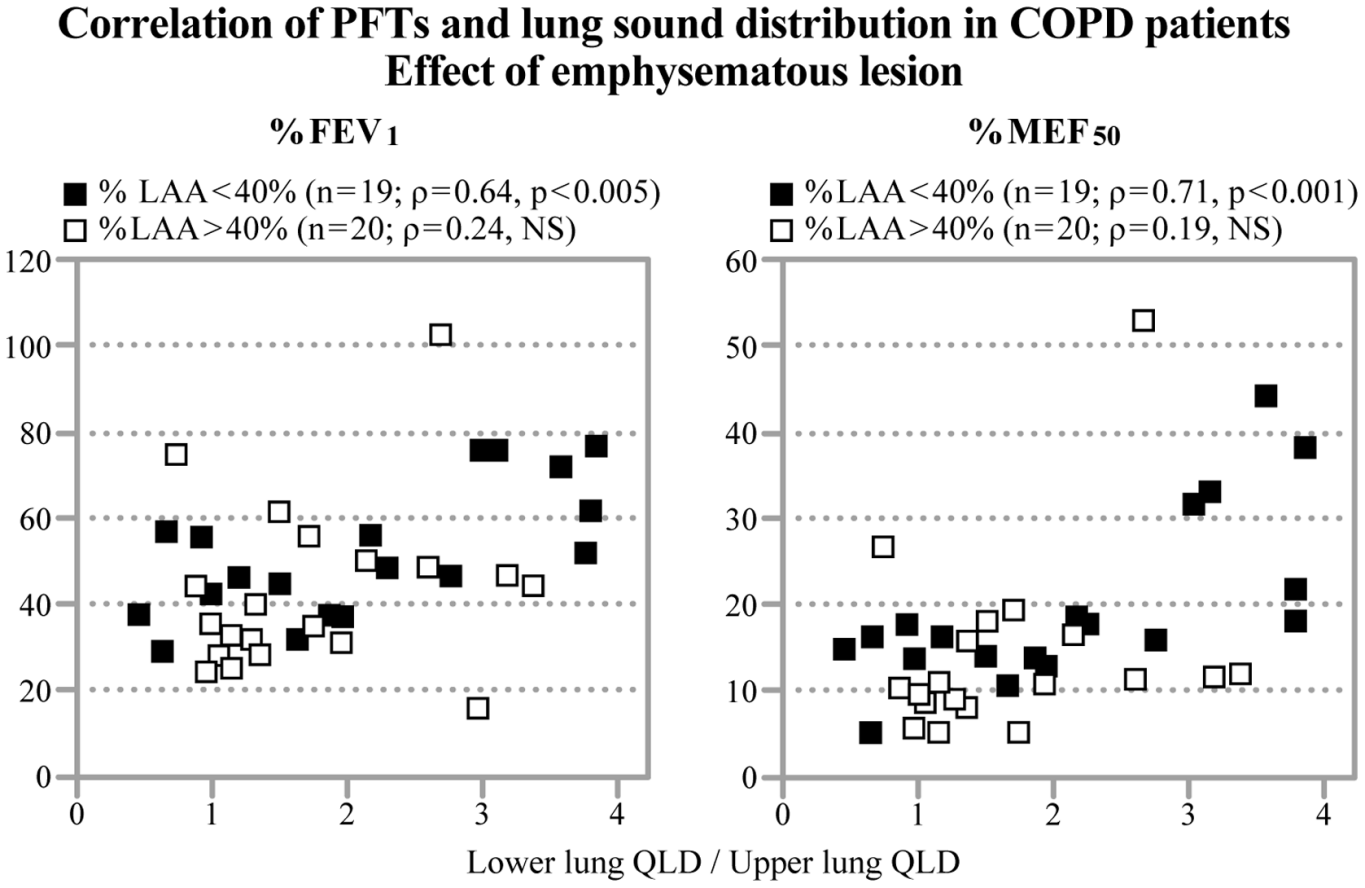
Correlation between PFTs and lung sound distribution in COPD patients and the effects of emphysematous lesions. In the less emphysematous group (%LAA <40%, n = 19), the relationship between Lower QLD/Upper QLD and obstructive changes (%FEV_1_; r = 0.64 [p<0.005], %MEF_50_; r = 0.71 [p<0.001]) were stronger than in the emphysema-dominant group (%LAA >40%, n = 20), in which these correlations were not observed.

To evaluate the effect of emphysematous lesions on the relationships between lung sound distribution and obstructive changes, we analyzed CT images for 39 COPD patients. The mean value for the %LAA was 36.7% (4.3% to 65.2%), and there were 20 emphysema-dominant COPD patients (%LAA>40%) and 19 less emphysematous COPD patients (%LAA<40%). In the less emphysematous group, the relationship between Lower QLD/Upper QLD and obstructive changes was stronger (%FEV_1_; ρ = 0.65 [p<0.005], FVC/FEV_1_; ρ = 0.74 [p<0.001],%MEF_50_; ρ = 0.71 [p<0.001]). However, these correlations were not observed in the emphysema-dominant group (Table 3, Figure 2).

**Figure 3:**
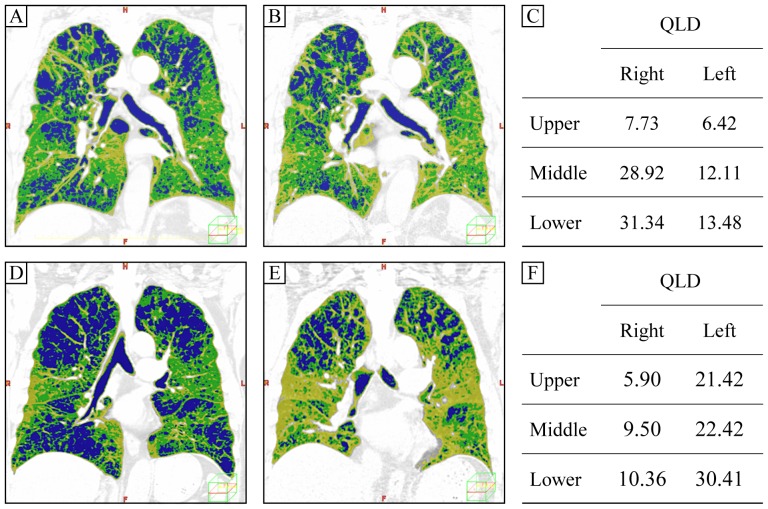
CT and lung sound distribution findings for 2 representative cases of upper-lung-dominant emphysema. Figure 3A to C: This subject was an 80-year-old male with GOLD III airflow limitation and an average %LAA of 47.6%. The inspiration CT showed upper-lung-dominant centrilobular emphysema (Figure 3A). After expiration, lower-lung volume tended to decrease more than upper-lung volume (Figure 3B). In this case, the lung sound intensity was lower-lung-dominant, and Lower QLD/Upper QLD was similar to the average value of healthy subjects (Figure 3C; Lower QLD/Upper QLD  = 3.17). Figure 3D to F: This subject was a 72-year-old male with GOLD II airflow limitation and an average %LAA of 44.1%. Inspiration CT showed upper-lung-dominant centrilobular emphysema (Figure 3D). After expiration, the decreases in the upper- and lower-lung volumes were almost equal (Figure 3E). In this case Lower QLD/Upper QLD was lower than average values in the COPD patients (Figure 3F; Lower QLD/Upper QLD  = 1.49).

The effect of emphysematous lesions on lung sound distribution was different in each patient. Figure 3 shows CT findings of two representative patients with upper-lung-dominant emphysema. One patient (Figure 3A–C) was an 80-year-old male with GOLD III airflow limitation (predicted FEV_1_ = 46.6%, predicted FVC = 110.1%) and an average %LAA of 47.6%. The inspiration CT (Figure 3A) showed upper-lung-dominant centrilobular emphysema (%LAA of upper lung field; Right  = 58.75%, Left  = 59.22, %LAA of lower lung field: Right  = 33.74%, Left  = 32.68%). After expiration, the lower-lung volume tended to decrease to a greater extent than the upper-lung volume (Figure 3B). In this case, the lung sound intensity was lower-lung-dominant and Lower QLD/Upper QLD was similar to the average values of healthy subjects (Figure 3C; Upper QLD  = 14.15%, Lower QLD  = 44.82%, Lower QLD/Upper QLD  = 3.17).

Figure 3D–F shows the CT and QLD findings of a subject with upper-lung-dominant emphysema. This subject was a 72-year-old male with GOLD II airflow limitation (predicted FEV_1_ = 61.4%, predicted FVC = 106.4%) and an average %LAA of 44.1%. The inspiration CT showed upper-lung-dominant centrilobular emphysema (%LAA of upper lung field; Right  = 64.11%, Left  = 54.88, %LAA of lower lung field: Right  = 35.27%, Left  = 29.42%). After expiration, the decreases in upper- and lower-lung volumes were nearly equal (Figure 3E). In this case, Lower QLD/Upper QLD was lower than the average of COPD patients (Figure 3F; Upper QLD  = 27.32%, Lower QLD  = 40.77%, Lower QLD/Upper QLD  = 1.49).

## Discussion

In this study, we found that Lower QLD/Upper QLD in COPD patients was significantly lower than in healthy subjects. We also found that significant correlations between obstructive changes and the upper-lung-dominant distribution of lung sound intensities were present in the COPD patients. These correlations, however, were not observed in emphysematous patients whose%LAA was over 40%. To the best of our knowledge, this is the first study to demonstrate that the correlation between lung sound distribution and the obstructive changes in pulmonary function was considerably affected by the presence of emphysematous lesions.

In severe COPD patients, movement of the abdominal ribcage and diaphragm are restricted by hyperinflation. A weak correlation between FEV_1_ and the displacement of the lateral rib margin has been observed in severe COPD patients [18]. Furthermore, accessory respiratory muscles, such as the sternocleidomastoid and scalene muscles, are recruited to raise the rib cage during tidal breathing in patients with severe obstructive pulmonary function. Under these circumstances, the regional ventilation may shift from the lower- to the upper-lung field as a function of the degree of hyperinflation, and the lung sound intensity may reflect these changes. Shi et al. reported that lung sound analysis using VRI was capable of detecting regional ventilation distribution under carefully controlled laboratory conditions [12]. In this study, we found that the ratio of lower- to upper-lung sound intensity in COPD patients was significantly lower than in healthy subjects, and that this ratio decreased according to the severity of the obstructive changes.

From the results of our previous study, we assumed that the distribution of emphysematous lesions might influence the regional lung ventilation and sound distribution [13]. In this study, a correlation between lung sound distribution and pulmonary function was not observed in emphysema-dominant patients. We found that lung sound distribution in emphysema-dominant patients varied. For example, in 8 upper-lung-dominant emphysema patients who had averaged %LAA values that were >40%, the range of the Lower QLD/Upper QLD values was from 0.73 to 3.17. On the other hand, in 3 lower-lung-dominant emphysema patients who had averaged %LAA values that were >40%, the range of Lower QLD/Upper QLD values were from 1.33 to 3.35.

There are several possible reasons for the diverse lung sound distribution in the emphysematous patients. First, the distribution of emphysematous lesions is heterogeneous. Because the changes in lung structure that occur in this disease affect the amplitude and sound transmission from the airways to the chest surface [19], lung sound intensity in the region of the emphysematous and non-emphysematous lesions is thought to be quite different. Second, the regional ventilation of each emphysematous lesion also differs. Using Dual Xenon CT, Park et al demonstrated that emphysematous lesions show different ventilation patterns, even in the same patients [20]. Emphysematous lesions with no or little regional ventilation may create a very weak lung sound and may even block sound transmission. On the other hand, ventilated emphysematous lesions may create respiratory sounds proportional to the level of ventilation. If the relationship between the regional lung sound intensity and ventilation in emphysematous lesions was determined, lung sound analysis could be used as a non-invasive bed-side physiological assessment tool for detection of emphysematous lesions in interventional treatments such as bronchoscopic lung volume reduction [21]–[23]. There is a possibility that a strong lung sound in the presence of emphysematous lesions might represent abundant collateral ventilation, which is a diagnostic criterion for the exclusion of patients from bronchoscopic lung volume reduction using one-way valves [23], [24]. Since we could recruit only a few patients who had been examined by both inspiratory and expiratory CT, further study to evaluate the relationship between regional ventilation and lung sound intensity in emphysematous lesions is required. Third, the severity of small airway obstruction may vary within each patient. In some cases of heterogeneous emphysema, small airway disease seems to contribute to an obstructive change similar to that of the emphysematous lesions. Research on airway properties (i.e. airway dimensions and airway wall thickness) and lung sound distribution will provide valuable insights in this field. For these aforementioned reasons, it may be difficult to find a correlation between obstructive changes and the distribution of the lung sound intensity in emphysema-dominant COPD patients.

In the less emphysematous group, the lung sound intensity moderately to strongly correlate with the obstructive changes. In these circumstances, lung sound analysis may be beneficial to the assessment of outcome after intervention. COPD treatment targeting the amelioration of hyperinflation may improve diaphragm movement by deflating the lungs, which may lead to increased ventilation and an airflow shift to the lower lung field. This phenomenon is reflected by a change in the lung sound distribution.

There have been extensive developments in functional imaging related to regional lung function, such as the Xenon ventilation CT [20] and hyperpolarized MRI [25]. These innovative and sophisticated methods will provide valuable insights into regional lung function in COPD patients. The correlation of these insights with the evaluation of acoustic findings using computer assisted lung sound analysis will enhance the value of lung auscultation in COPD management. Lung auscultation is still an essential part of the physical examination, which brings clinical information about the respiratory system quickly, easily, and cost-effectively [26].

There were some limitations to this study. First, the attachment of sensor to the bony posterior chest wall was difficult, which resulted in the exclusion of patients with a BMI of 19 or less. Low body weight is common in Japanese COPD patients, and further improvements in sensor attachment technology are expected. Second was the absence of female COPD patients in this study due to the low number of female COPD patients at our institution. Because there are differences in lung sound distribution between males and females in healthy subjects [17], further study including female COPD patients is needed. Third, we were unable to recruit a sufficient number of age-matched healthy smokers. Since we could not find an effect of age on Lower QLD/Upper QLD in healthy subjects, we believe the difference in age between healthy subjects and COPD patients did not influence the results of Lower QLD/Upper QLD analysis of this study.
